# Systemic and tumor level iron regulation in men with colorectal cancer: a case control study

**DOI:** 10.1186/1743-7075-11-21

**Published:** 2014-05-13

**Authors:** Cenk K Pusatcioglu, Elizabeta Nemeth, Giamila Fantuzzi, Xavier Llor, Sally Freels, Lisa Tussing-Humphreys, Robert J Cabay, Rose Linzmeier, Damond Ng, Julia Clark, Carol Braunschweig

**Affiliations:** 1 Division of Gastroenterology, Hepatology, and Nutrition, Ann & Robert H. Lurie Children’s Hospital of Chicago, 225 E Chicago Ave, Chicago, IL 60611, USA; 2 Department of Medicine, University of California, Los Angeles, 10833 Le Conte Ave, Los Angeles, CA 90095, USA; 3 Department of Kinesiology and Nutrition, University of Illinois at Chicago, 1919 W Taylor St, Chicago, IL 60612, USA; 4 Section of Digestive Disease and Nutrition, University of Illinois at Chicago, 840 S Wood St, Chicago, IL 60612, USA; 5 Division of Epidemiology and Biostatistics, University of Illinois at Chicago, 1603 W Taylor St, Chicago, IL 60612, USA; 6 Department of Medicine, University of Illinois at Chicago, 1747 W Roosevelt Rd, Chicago, IL 60608, USA; 7 Department of Pathology, University of Illinois at Chicago, 840 S Wood St, Chicago, IL 60612, USA

**Keywords:** Iron metabolism, Hepcidin, Inflammation, Anemia, Colorectal cancer

## Abstract

**Background:**

Increased cellular iron exposure is associated with colorectal cancer (CRC) risk. Hepcidin, a liver peptide hormone, acts as the primary regulator of systemic iron status by blocking iron release from enterocytes into plasma. Concentrations are decreased during low iron status and increased during inflammation. The role of hepcidin and the factors influencing its regulation in CRC remains largely unknown. This study explored systemic and tumor level iron regulation in men with CRC.

**Methods:**

The participants were 20 CRC cases and 20 healthy control subjects. Colonic tissue (adenocarcinoma [cases] healthy mucosa [controls]) was subjected to quantitative PCR (hepcidin, iron transporters and IL-6) and Perls’ iron staining. Serum was analyzed using ELISA for hepcidin, iron status (sTfR) and inflammatory markers (CRP, IL-6, TNF-α). Anthropometrics, dietary iron intake and medical history were obtained.

**Results:**

Cases and controls were similar in demographics, medication use and dietary iron intake. Systemically, cases compared to controls had lower iron status (sTfR: 21.6 vs 11.8 nmol/L, p < 0.05) and higher marker of inflammation (CRP: 8.3 vs 3.4 μg/mL, p < 0.05). Serum hepcidin was mildly decreased in cases compared to controls; however, it was within the normal range for both groups. Within colonic tissue, 30% of cases (6/20) presented iron accumulation compared to 5% of controls (1/20) (χ^2^ = 5.0; p < 0.05) and higher marker of inflammation (IL-6: 9.4-fold higher compared to controls, p < 0.05). Presence of adenocarcinoma iron accumulation was associated with higher serum hepcidin (iron accumulation group 80.8 vs iron absence group 22.0 ng/mL, p < 0.05).

**Conclusions:**

While CRC subjects had serum hepcidin concentrations in the normal range, it was higher given their degree of iron restriction. Inappropriately elevated serum hepcidin may reduce duodenal iron absorption and further increase colonic adenocarcinoma iron exposure. Future clinical studies need to assess the appropriateness of dietary iron intake or iron supplementation in patients with CRC.

## Background

Excessive body iron levels are associated with increased risk for colorectal cancer (CRC) [1-6]. This is due to the high oxidative potential of iron which can result in the formation of reactive oxygen species and mutate the DNA of key genes involved in cell proliferation [2]. Additionally, since iron is required for cell proliferation, increased iron levels could contribute to tumor promotion [1].

Iron levels and its tissue distribution in the body are regulated by hepcidin, a hepatic-derived systemic iron regulatory hormone [7-9]. Hepcidin is increased by inflammation and decreased by iron insufficiency and erythropoiesis [10-12]. Mechanistically, hepcidin controls iron release into plasma by degrading the iron exporter ferroportin (FPN) in cells that handle iron, including intestinal enterocytes, hepatocytes, and macrophages [13]. Thus, when concentrations of hepcidin are elevated, FPN expression is low, resulting in reduced dietary iron absorption and impaired mobilization from stores.

Few studies have assessed the role of hepcidin and FPN in cancer [14,15]. Serum hepcidin levels were found to be elevated in some hematologic or nonhematologic cancers, likely because of the presence of inflammation, but the pathophysiological relevance of these findings is unknown [16,17]. For FPN, it was reported that patients with breast cancer had decreased tumor expression of FPN protein compared to non-involved tissue, and that tumor FPN mRNA levels were negatively correlated with advanced staging [14]. This suggested that iron retention by breast cancer cells may affect cancer progression.

Hepcidin may be elevated in persons with CRC due to cancer-induced inflammation [18,19]. However, because CRC patients frequently have anemia or low iron status, hepcidin levels may also be decreased [20,21]. Evaluation of the involvement of hepcidin in regulating systemic and tumor level iron metabolism in CRC is limited to one study [15]. Ward et al. reported that systemic hepcidin is elevated with advanced cancer staging [15]. Additionally, they demonstrated that mRNA expression of hepcidin is detected within a subset (34%) of colonic tumors compared to healthy non-involved mucosa. In a complementary study by Brookes et al., increases in iron acquisition proteins (Divalent metal transporter-1, DMT-1; Transferrin receptor-1, TfR1) and decreases in proteins related to cellular iron efflux (FPN; Hephaestin, Heph) were noted in colonic tumors when compared to non-involved mucosa [1]. The authors suggested that these alterations in iron transport may explain the iron sequestration commonly observed in colonic tumors [1]. What remains unknown is hepcidin’s role in regulating colonocyte iron transport and whether it contributes to tumor iron accumulation in persons with CRC.

The purpose of this study was to examine simultaneously systemic and tumor iron status and their regulation in men with CRC compared to controls. This was assessed by measuring: (i) hepcidin, iron status and markers of inflammation in serum and (ii) hepcidin, expression of iron transporters (DMT-1, FPN), inflammation and iron accumulation in colonic mucosa. We hypothesized that CRC would be associated with higher levels of hepcidin in serum and tumor compared to controls and that hepcidin levels would be correlated with markers of inflammation and mucosal iron accumulation.

## Methods

### Ethics

All subjects signed an informed consent and the study procedures were approved by the University of Illinois at Chicago Institutional Review Board.

### Study population and characteristics

Study subjects were recruited from patients scheduled for colonoscopies due to abdominal pain, bloating, change in bowel movements or for CRC screening at the University of Illinois at Chicago and John H. Stroger Jr. Cook County Hospital between May 2011 and June 2012. "Cases" were classified as newly diagnosed CRC with adenocarcinoma based on pathology reports for their tumor biopsies; "controls" were selected from the subjects with healthy colonic mucosa (absence of adenomatous polyps or GI abnormalities). Cases and controls (n = 20/group) were matched to have a similar difference within each pair for age (within 5 years), body mass index (BMI) (within 4 units) and waist circumference (within 5 cm). Due to gender-specific variation in reference ranges for iron parameters, participation was restricted to males. Additional exclusions included medical conditions that could affect iron status such as gastrointestinal bleeding, hemochromatosis, history of inflammatory bowel disease or infection.

Following informed consent, questionnaires for basic demographic information, health history, medication and supplement use and alcohol consumption were administered by a research team member. The Block Brief 2000 food frequency questionnaire (FFQ) was used to assess usual dietary intake over the previous 12 months [22]. Height was measured with a stadiometer to nearest 0.1 cm and weight using a balance beam scale to nearest 0.1 kilograms with subjects wearing a hospital gown. Waist circumference was measured with a flexible tape (AccuFitness, Greenwood Village, CO) at the midpoint between the ribs and iliac crest, to the nearest 0.1 cm. Body mass index was calculated as weight in kilograms divided by height in meters squared. CRC staging (0-IV) based on tumor size, lymph nodes affected and metastasis (TNM) was classified using the American Joint Committee on Cancer (AJCC) criteria [23].

### Laboratory assays

All blood samples were collected after a minimum 12 hour fast following the endoscopy or prior to surgical intervention. A separate analysis did not reveal any differences in serum parameters by blood collection type. Colonic adenocarcinoma tissue was obtained from cases at the time of surgical resection. Healthy colonic mucosa from the descending colon in the controls was obtained using standard sized biopsy forceps during colonoscopy.

### Serum parameters

Systemic iron regulation was assessed by serum hepcidin and iron status by serum transferrin receptor (sTfR), which primarily reflects erythroid iron demand. Serum transferrin receptor is not influenced by acute or chronic inflammation and can help differentiate between iron-related disorders [24]. Elevated sTfR is indicative of iron-deficient erythropoiesis [25]. Anemia was examined by hemoglobin (Hb). Inflammation was evaluated via quantification of C-reactive protein (CRP), interleukin-6 (IL-6) and tumor necrosis factor-alpha (TNF-α).

Serum hepcidin was measured by competitive enzyme-linked immunosorbent assay (c-ELISA) (Intrinsic LifeSciences, La Jolla, CA). For healthy men with normal iron status, the range for this assay is 29–254 ng/mL [8]. Serum transferrin receptor was measured using the Quantikine IVD Human sTfR Immunoassay ELISA (R&D Systems, Minneapolis, MN; normal reference range 8.7-28.1 nmol/L). Hemoglobin was obtained from the medical chart and anemia was defined as <12 g/dL [21]. CRP, IL-6 and TNF-α were measured using R&D Systems Quantikine ELISA kits (R&D Systems, Minneapolis, MN).

### Tissue specimen

A portion of colonic tissue was placed in formalin and paraffin-embedded for histological analysis. The remaining portion of the colonic tissue was placed in RNAlater (Ambion, Austin, TX) and stored at -80°C for gene expression (mRNA) analysis.

### Real time polymerase chain reaction (RT-PCR)

Total RNA was extracted from colonic mucosa sections using the Maxwell 16 System (Promega, Fitchburg, WI). The complementary DNA was synthesized from the RNA using iScript™ cDNA Synthesis Kit (BioRad, Hercules, CA). Gene expression (mRNA) of divalent metal transporter-1 (DMT-1), ferroportin (FPN), hepcidin and IL-6 were measured quantitatively by RT-PCR (For primers used see Additional file 1) using SsoAdvanced SYBR Green Supermix (BioRad). Amplifications were performed at 57°C for 40 cycles using the C1000 Touch (BioRad) and data analyzed using the CFX Manager software (BioRad). Results were normalized to reference genes Glyceraldehyde 3-phosphate dehydrogenase (GADPH) and β-actin to obtain ΔCt values (ΔCt = Ct[reference] - Ct[target]). As both GADPH and β-actin showed equivalent results, we used the average of the two values for the final representation. Fold change in mRNA expression in cases compared to controls was calculated using 2^ΔΔCt^, where ΔΔCt = average ΔCt for cases - average ΔCt for controls. Statistical comparison was performed using raw ΔCt values (Additional file 2).

### Iron staining

Tissue iron accumulation was measured qualitatively by Perls’ Prussian blue staining. The tissue section was treated with dilute hydrochloric acid to release ferric ions from binding proteins. These ions then reacted with potassium ferrocyanide to produce an insoluble blue compound (the Prussian blue reaction). The grading of iron staining was performed by a pathologist blinded to the experimental group and reported dichotomously as presence or absence of iron accumulation (+/-).

### Statistical analysis

Prior to analysis, all variables were assessed for normality and presence of outliers. Descriptive statistics included mean, standard deviation (SD), median and interquartile range (IQR) for continuous variables and frequencies for categorical variables. Difference between cases and controls was assessed by student’s paired *t*-test or non-parametric Wilcoxon signed-rank test for continuous variables and McNemar’s test for categorical variables. Non-parametric Spearman’s Rank correlation coefficient was used to explore bivariate relationships. Multivariable linear regression was performed to adjust for confounders. All analyses were performed using SAS version 9.3 (Cary, North Carolina). P-values were two-sided and the statistical significance level was defined as p < 0.05.

## Results

Subject characteristics are presented in Table 1. By design cases and controls were similar in age, BMI and waist circumference. Race/ethnicity, dietary iron intake (heme and non-heme food and supplemental sources), medication use and alcohol consumption were also similar between cases and controls.

**Table 1:** Subject characteristics between cases and controls

	**Cases (n = 20)**	**Controls (n = 20)**	P-value^*^
**Age**	61.0 (8.0)	57.5 (12.0)	0.25
**BMI**	25.7 (5.0)	26.9 (7.3)	0.38
**WC(cm)**	100.4 (11.5)	103.3 (18.2)	0.49
**Race (%)**			
**African American**	50%	75%	0.16
**White**	30%	5%	
**Hispanic**	5%	10%	
**Asian**	15%	10%	
**Dietary iron (grams/day)**			
**Heme**	6.7 (4.3)	7.0 (4.3)	0.99
**Non-heme**	0.67 (0.7)	0.66 (0.64)	0.99
**Alcohol (grams/day)**	0.23 (2.0)	1.0 (2.1)	0.46
**Medications use (yes)**			
**Aspirin**	40%	65%	0.16
**NSAIDs**	50%	40%	0.53
**COX-2 inhibitors**	10%	5%	0.56
**Cancer staging**^**†**^			
**I**	15%		
**II**	40%		
**III**	30%		
**IV**	15%		

Values shown as median (IQR), or percentage.

*p-value of difference between cases and controls using Wilcoxon signed-rank test or McNemar’s test as appropriate.

^**†**^American Joint Committee on Cancer (AJCC) criteria.

BMI, body mass index; WC, waist circumference.

### Systemic iron regulation and inflammation

Systemic iron status and inflammatory parameters are presented in Table 2. Cases had significantly lower Hb and higher sTfR compared to controls (p < 0.05), indicative of iron-restricted erythropoiesis. Serum hepcidin was mildly decreased in cases, likely as a response to the increased erythroid iron demand (as reflected by the elevated sTfR). However, considering that hepcidin concentrations were still in the normal range for cases, hepcidin may be elevated given their degree of iron restriction. Hepcidin is known to be low or undetectable in individuals with insufficient iron status [8]. Cases also had significantly elevated CRP (p < 0.05) and a trend toward increased IL-6 concentrations compared to controls, indicating the presence of mild inflammation in these patients. This may stimulate hepcidin production and counterbalance the suppressive effect of iron-restricted erythropoiesis on hepcidin.

**Table 2:** Iron status and markers of inflammation in serum between cases and controls

	**Cases (n = 20)**	**Controls (n = 20)**	P-value*
**Hb (g/dL)**^**†**^	11.7 (2.9)	13.2 (1.3)	0.01
**sTfR (nmol/L)**^**†**^	21.6 (17.0)	11.8 (6.1)	0.01
**Hepcidin (ng/mL)**^**†**^	58.6 (62.1)	96.0 (34.1)	0.10
**CRP (μg/mL)**	8.3 (3.4)	3.4 (4.7)	0.002
**IL-6 (pg/mL)**	2.7 (2.2)	1.9 (1.3)	0.06
**TNF-α (pg/mL)**	0.32 (0.27)	0.24 (0.36)	0.62

Values shown as median (IQR).

*p-value of difference between cases and controls using Wilcoxon signed-rank test.

†Reference range for healthy men [9]. Hb, 13.5-17.5 g/dL; sTfR, 8.7-28.1 ng/mL; Hepcidin, 29–254 ng/mL.

Hb, hemoglobin; sTfR, serum transferrin receptor; CRP, c-reactive protein; IL-6, interleukin-6; TNF-α, tumor necrosis factor-alpha.

Associations between serum hepcidin and iron status and inflammatory parameters were explored with Spearman correlation coefficients. In controls, serum hepcidin was correlated with Hb (r = 0.54; p = 0.01) as expected, and no associations were found with sTfR or inflammatory markers (CRP, IL-6, TNF-α), which were in the normal range. In cases, serum hepcidin was not correlated with any of the parameters (Hb, sTfR or the inflammatory markers). Additionally, cancer staging did not change serum hepcidin concentrations (Stage IV: median 64.4 (IQR 249.1) versus Stage I: 72.8 (IQR 28.7) ng/mL p = 1.0). Since our lab previously demonstrated that obesity-induced inflammation is associated with elevated serum hepcidin levels, we stratified cases and controls by obesity status (obese ≥ 102 cm, lean < 102 cm) [26,27]. Within cases, circulating markers of inflammation and serum hepcidin did not differ by obesity status (data not shown). Obese controls were more inflamed (CRP, IL-6) compared to lean; however, serum hepcidin concentrations were similar between the groups.

### Iron transporters in colonic mucosa

Gene expression (mRNA) of hepcidin, iron transporters (DMT-1 and FPN) and the pro-inflammatory protein IL-6 in colonic mucosa are presented in Table 3. Expression of DMT-1 and FPN were similar between cases and controls. Although cases had a 2.9-fold lower expression of hepcidin compared to controls (p < 0.05), overall both groups expressed very low hepcidin mRNA in the colonic mucosa (close to the limit of detection by qPCR). Cases had a 9.4-fold higher expression of IL-6 compared to controls (p < 0.05), confirming the inflammatory nature of the tumor. Correlation analyses did not reveal significant associations between expression of the serum and colonic markers of iron regulation, iron transport or inflammation in cases or controls (data not shown).

**Table 3:** **mRNA expression of iron transporters and inflammatory proteins in colonic tissue of colorectal cancer cases and controls using 2**^**ΔΔCt **^**conversion**

	**Cases (n = 20)**	**Controls (n = 20)**	P-value*
**DMT-1**	1.12	1.0	0.59
**FPN**	0.81	1.0	0.59
**Hepcidin**	0.35	1.0	0.001
**IL-6**	9.38	1.0	0.0002

Values shown as relative expression in cases compared to controls, i.e. reference.

*p-value of difference between cases and controls using student’s paired *t*-test.

DMT-1, divalent metal transporter-1; FPN, ferroportin; IL-6, interleukin-6.

### Iron accumulation in colonic mucosa

**Figure 1:**
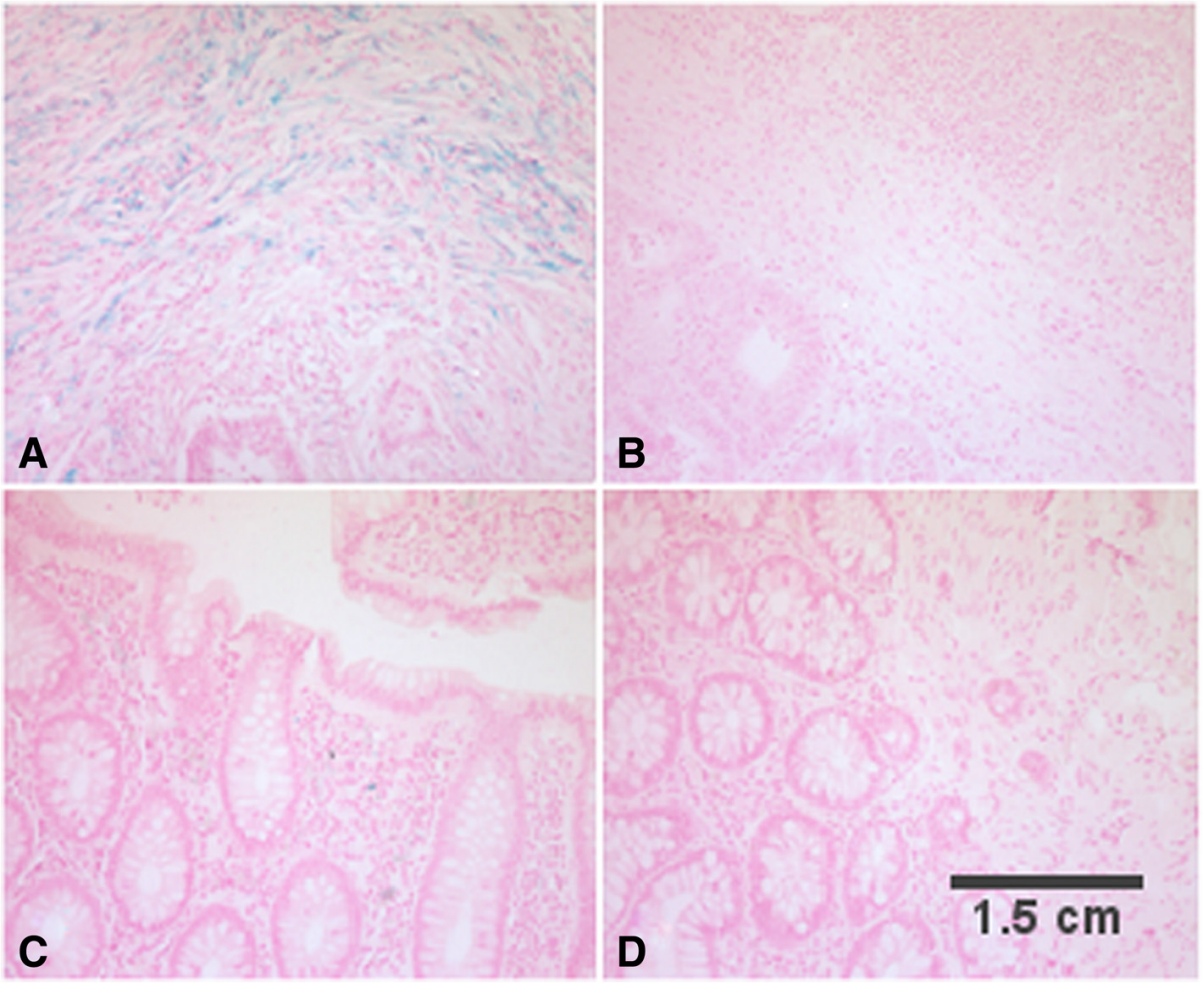
**Iron staining in colonic tissue between cases and controls.** Sections of colonic tissue (adenocarcinoma for cases, healthy mucosa for controls) were tested for presence of iron accumulation using the Perls’ Prussian blue staining. Cases with presence **(A)** and absence **(B)** of iron accumulation were compared to controls with presence **(C)** and absence of iron accumulation **(D)** and presented as 20X magnification. There was more iron accumulation detected in cases than controls (n = 6/20; 30%; n = 1/20; 5%; McNemar’s test: *χ*^2^ = 5.0; p < 0.05).

To determine if CRC was associated with greater tissue iron accumulation, adenocarcinoma from cases and healthy colonic mucosa from controls was examined using Perls’ Prussian blue stain. Iron accumulation was present in more cases than controls (n = 6/20; 30%; n = 1/20; 5%, χ^2^ = 5.00; p < 0.05). Illustrative Perls’ stains are shown in Figure 1 (cases A-B, controls C-D). When cases were dichotomized by presence/absence of iron accumulation (+/-), cases with iron accumulation had higher serum hepcidin compared to the cases without iron accumulation (p < 0.05) (Table 4). However, after adjusting for Hb, differences in serum hepcidin between these subgroups became non-significant. This suggests that systemic iron sufficiency may have contributed to the higher serum hepcidin observed in the iron accumulation (+) group given that hepcidin is increased when iron stores are adequate. In support, there was a trend for lower sTfR in the iron (+) group, demonstrating iron bioavailability. Differences in systemic inflammatory markers (CRP, IL-6 or TNF-α) between cases with different iron accumulation (+/-) were non-significant. Additionally, no differences were observed for tissue level (mRNA expression for DMT-1, FPN, hepcidin, IL-6) parameters or with cancer staging (data not shown).

**Table 4:** Iron status and markers of inflammation in serum between cases with colonic iron presence (+) and without iron presence (-)

	**Iron (+)**	**Iron (-)**	P-value*	**P-value†**
	**(n = 6)**	**(n = 14)**		
**Hb (g/dL)**	12.8 (2.0)	11.2 (3.4)	0.20	-
**sTfR (nmol/L)**	11.0 (14.3)	22.2 (18.0)	0.11	0.32
**Hepcidin (ng/mL)**	80.8 (28.6)	22.0 (48.4)	0.02	0.12
**CRP (μg/mL)**	7.7 (3.4)	7.9 (3.4)	0.43	0.31
**IL-6 (pg/mL)**	2.5 (3.0)	2.7 (2.0)	0.99	0.86
**TNF-α (pg/mL)**	0.27 (0.3)	0.38 (0.3)	0.46	0.41

Values shown as median (IQR).

*p-value of difference between iron(+) and iron (-) using non-parametric Mann–Whitney *U* test.

†p-value of difference between iron(+) and iron (-) adjusting for Hb with multiple linear regression.

Hb, hemoglobin; sTfR, serum transferrin receptor; CRP, c-reactive protein; IL-6, interleukin-6; TNF-α, tumor necrosis factor-alpha.

## Discussion

Individuals with CRC frequently have anemia or low iron status which may, at least in part, be due to an inflammatory response termed the anemia of chronic disease (ACD) [18,20,21,28,29]. The ACD is thought to manifest through increased hepatic production of the iron regulatory protein hepcidin, which can down-regulate the iron exporter FPN [10,13,30]. Reduction of FPN limits iron flow into circulation from both the diet and storage sites and promotes tissue iron sequestration. Beyond impaired iron status and red blood cell production, the ACD may be relevant for persons with CRC given that hepcidin-induced reductions in dietary iron absorption in the proximal gut may increase colonic iron exposure. Colonic tumors may benefit from this alteration since iron is a substrate for cell proliferation, cancer progression and metastasis [1,2,5,6]. The primary aims of this case–control study were to simultaneously examine systemic and tumor level iron status and regulation in men with CRC compared to controls and determine if systemic or tumor level hepcidin expression was associated with tumor iron accumulation.

We found cases had significantly more iron-restricted erythropoiesis based on Hb and sTfR compared to controls. We could not differentiate whether this mild anemia was due to inflammation or also to frank iron deficiency. Often, gastrointestinal bleeding can contribute to iron deficiency in CRC; however, we excluded persons with known gastrointestinal bleeding, suggesting other factors contributed to the iron restriction observed in cases [29,31]. Typically when iron deficiency is present, serum hepcidin concentrations are undetectable [8]. Indeed, cases had lower serum hepcidin compared to controls; however, this difference was not significant and was within the normal range. This suggests hepcidin levels were inappropriately elevated given the patients’ iron-restricted erythropoiesis. Hepcidin expression is simultaneously regulated by inflammation, iron stores and erythropoiesis [7,10]. Further, hepcidin levels are ultimately determined by the relative strength of these opposing stimuli [12,32]. In contrast to controls where serum hepcidin was correlated with Hb, serum hepcidin within cases was not significantly correlated with Hb suggesting that multiple signals may be influencing its production. Significantly greater systemic and colonic mucosal inflammation was observed in cases compared to controls which may have provided the necessary stimuli to promote increased hepatic hepcidin production, despite elevated sTfR and low iron status. This phenotype, in which serum hepcidin is simultaneously regulated by low iron status and inflammation, has been previously reported in morbid obesity [26,33,34]. Similar to our cases, obese participants in these studies presented with low iron status, elevated systemic inflammation and serum hepcidin concentrations within the normal range [26]. Like obesity, the iron profile observed in our cases is consistent with a mixed anemia for which hallmarks of the ACD and iron deficiency coexist [9]. This phenotype allows for the mobilization of iron from body stores but impairs iron repletion as a result of reduced dietary iron absorption [35]. Therefore, over time, inflammation-induced chronically elevated hepcidin would precipitate cellular iron depletion as body iron losses exceed dietary absorption effort [27,36]. Importantly, this type of mixed anemia has the potential to increase gastrointestinal iron exposure in persons with CRC.

We assessed colonic mucosal iron transport and regulation and found no statistical difference in expression of DMT-1 or FPN between cases and controls. Directionally our DMT-1 and FPN expression were similar to those of Brookes et al. [1]. They reported increased colonocyte expression of iron influx (DMT-1) and decreased iron efflux (FPN) proteins in CRC compared to controls. This suggests that CRC tissue is associated with greater colonic adenocarcinoma iron uptake and sequestration compared to healthy mucosa. We observed very low hepcidin expression in the colonic tumors and healthy mucosa. This is not unexpected given that hepcidin is primarily produced in hepatocytes. In our previous analysis, abdominal subcutaneous and visceral adipose tissue also expressed very low mRNA expression compared to the liver [26]. Very little is known regarding the role of extra-hepatic hepcidin in systemic and tissue-level iron regulation. Our findings indicate that colonic hepcidin has a minimal role in tumor iron regulation. Ward et al*.* reported elevated tumor hepcidin expression within 34% of cases compared to non-involved healthy mucosa; however, other factors that regulate hepcidin expression including inflammation, iron status and dietary iron intake were not examined, limiting interpretations of their findings [15]. Given that few studies have examined the role of extra-hepatic hepcidin in regulating systemic or tissue-level iron metabolism, further investigations are warranted to understand mechanisms related to iron metabolism in colonic tumors [15,37-39].

We observed significantly more cases (30%) had detectable iron accumulation compared to controls (5%). Perls’ staining has relatively low sensitivity. We speculate iron accumulation would have been much more prevalent in cases had we used a more sensitive assessment method. Brookes et al*.* used DAB-enhanced Perls’ methodology and detected visible iron accumulation in all (n = 20) of the CRC cases but not controls [1]. To further characterize the cases with iron accumulation, we examined systemic hepcidin and iron status parameters. The iron (+) group had elevated serum hepcidin compared to cases without iron accumulation. However, the iron (+) group was also more iron sufficient and had higher Hb. Given the small sample of cases with iron accumulation, it is hard to make any definitive conclusions. However, our data coupled with the report by Brookes et al*.* suggests that a subset of persons with CRC have increased expression of systemic hepcidin, which may precipitate greater intestinal iron exposure, and promote tumor iron retention [1]. In a murine model of CRC, animals had increased tumor growth when fed a high iron diet compared to a low iron diet [5]. Also, in persons with ulcerative colitis (UC), who have co-existing systemic and colonic inflammation and hepcidin-mediated dietary iron malabsorption, luminal iron exposure is associated with greater colonic inflammation and mucosal proliferation [40-42]. Therefore, studies examining the effect of dietary iron restriction in persons with CRC should be explored.

The link with hepcidin and excess tumor iron accumulation provides further evidence for its role in cancer. Only one other study has comprehensively explored the hepcidin-FPN axis and its relationship in cancer progression. Pinnix et al*.* using human breast cancer tissue found that low FPN and high hepcidin expression may enable rapid cell proliferation [14]. Unfortunately, the authors did not measure systemic hepcidin concentrations and future exploration should focus on its role in tumor iron retention in other epithelial cancers.

Our study had strengths and limitations. A significant strength was that we simultaneously measured hepcidin systemically and in colonic mucosa while accounting for several factors that can contribute to hepcidin regulation, including iron status, inflammation and dietary iron intake. Additionally, we included a metabolically healthy control group. However, this study had limitations, including a relatively small sample size. Therefore, when we stratified by presence of iron accumulation in the CRC cases, this may have reduced the statistical power to detect differences. Tissue analysis with adenocarcinoma in cases to healthy mucosa in a control group was not a direct comparison. Future studies should also include non-involved mucosa in cases to accurately describe our analysis among all groups. Due to limited tissue allocated for this study, we were unable to accurately measure protein expression of the colonic iron exporter via FPN. Ferroportin is post-translationally modified by hepcidin, which may have precluded us from observing a significant correlation between colonic FPN and hepcidin. Although we suggest dietary iron absorption may be impaired in persons with CRC, we did not examine dietary iron absorption in this study. Finally, our design included only males, thus generalizability to females may be limited.

## Conclusion

In summary, our study demonstrates for the first time that CRC in some men is associated with a mixed anemia pathology characterized by iron restricted erythropoiesis. We believe the simultaneous presence of inflammation (increases hepcidin) and iron insufficiency (suppresses hepcidin) in a subset of our men with CRC resulted in hepcidin concentrations inappropriately elevated given their depleted iron status. Further, these findings suggest that systemic hepcidin in some CRC cases may 1) decrease duodenal iron absorption resulting in low iron status and 2) contribute to excess colonic iron exposure and disease promotion. Given the high incidence of CRC and the accompanying low iron status, these findings could have significant clinical implications [21,43]. Future investigations into the risks and benefits of dietary iron intake and oral iron supplementation in persons with CRC are warranted.

## Competing interests

All of the authors declare they have no competing interests.

## Authors’ contributions

CKP and CB designed the research. CKP, JC and XL acquired the data. CKP, SF, RJC, RL and DN analyzed the data. CKP, SF, EN, GF, LTH, and CB interpreted the data. CKP wrote the manuscript draft. CKP, EN, GF, LTH, and CB critically revised the manuscript. All authors read and approved the final manuscript.

## Supplementary Material

Additional file 1 Primers used for mRNA analysis. Click here for file

Additional file 2 mRNA expression of iron transporters and inflammatory proteins in colonic tissue of colorectal cancer cases and controls using raw ΔCt values. Click here for file
