# Supplementary material for: Systemic and tumor level iron regulation in men with colorectal cancer: a case control study
Source: Nutr Metab (Lond). 2014 May 13;11:21. doi: 10.1186/1743-7075-11-21 (PMC4037273; doi:10.1186/1743-7075-11-21)
Supplement: Additional file 1 — Primers used for mRNA analysis. [file 1743-7075-11-21-S1.docx]

**Additional File 1** Primers used for mRNA analysis

human DMT-1 5’-TGAACCTAAAGTGGTCACGC -3’ (forward-1), 5’-GGGTATGAGAGCAAAGGGAAG-3’ (reverse-1), 5’-TCTACTTGGGTTGGCAATGTTT-3’ (forward-2), 5’-GGCTACCTGCAGAAGACAGACT-3’ (reverse-2)

human ferroportin 5’-CGGTGTCTGTGTTTCTGGTAGA-3’ (forward-1), 5’-CTGGGCCACTTTAAGTCTAGC-3’ (reverse-1), 5’-TTACCAGAA AAC CCC AGCTCTAG-3’ (forward-2), 5’-AGTCTTTCACACCCATTAGATGAG-3’ (reverse-2)

human hepcidin 5’-CTGCAACCCCAGGACAGAG-3’ (forward-1), 5’-TCTACGTCTTGCAGCACATCC-3’ (reverse-1), 5’-CTTCCCCATCTGCATTTTCTG-3’ (forward-2), 5’-CCAGCCATTTTATTCCAAGACC-3’ (reverse-2)

human IL-6 5’-CAC TCA CCT CTT CAG AAC GAA TTG A-3’ (forward-1), 5’-GCC ATC TTT GGA AGG TTC AGG TTG-3’ (reverse-1), 5’-CTT TTG GAG TTT GAG GTA TAC CTA GAG-3’ (forward-2), 5’-GTC AGG GGT GGT TAT TGC ATC TAG-3’ (reverse-2)

human GADPH 5’-GAA GGT GAA GGT CGG AGT CAA-3’ (forward), 5’-CAT GGG TGG AAT CAT ATT GGA ACA T-3’ (reverse)

human β-actin 5’- ACA CCT TCT ACA ATG AGC TGC GT-3’ (forward), 5’- GATAGCACAGCCTGGATAGC-3’ (reverse)
