# Supplementary material for: Systemic and tumor level iron regulation in men with colorectal cancer: a case control study
Source: Nutr Metab (Lond). 2014 May 13;11:21. doi: 10.1186/1743-7075-11-21 (PMC4037273; doi:10.1186/1743-7075-11-21)
Supplement: Additional file 2 — mRNA expression of iron transporters and inflammatory proteins in colonic tissue of colorectal cancer cases and controls using raw ΔCt values. [file 1743-7075-11-21-S2.docx]

**Additional File 2** mRNA expression of iron transporters and inflammatory proteins in colonic tissue of colorectal cancer cases and controls using raw ΔCt values


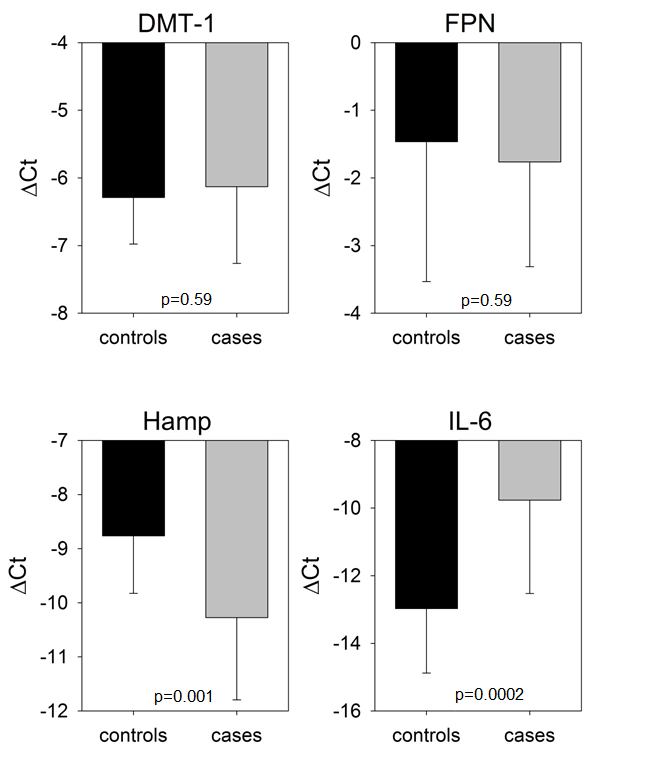


* p-value of difference between cases and controls using student’s paired *t*-test. *DMT-1, divalent metal transporter-1; FPN, ferroportin; Hamp, hepcidin; IL-6, interleukin-6.*
